# Interaction Studies of the Human and *Arabidopsis thaliana* Med25-ACID Proteins with the Herpes Simplex Virus VP16- and Plant-Specific Dreb2a Transcription Factors

**DOI:** 10.1371/journal.pone.0098575

**Published:** 2014-05-29

**Authors:** Ximena Aguilar, Jeanette Blomberg, Kristoffer Brännström, Anders Olofsson, Jürgen Schleucher, Stefan Björklund

**Affiliations:** 1 Department of Chemistry, Umeå University, Umeå, Sweden; 2 Department of Medical Biochemistry and Biophysics, Umeå University, Umeå, Sweden; Università degli Studi di Milano, Italy

## Abstract

Mediator is an evolutionary conserved multi-protein complex present in all eukaryotes. It functions as a transcriptional co-regulator by conveying signals from activators and repressors to the RNA polymerase II transcription machinery. The *Arabidopsis thaliana* Med25 (aMed25) ACtivation Interaction Domain (ACID) interacts with the Dreb2a activator which is involved in plant stress response pathways, while Human Med25-ACID (hMed25) interacts with the herpes simplex virus VP16 activator. Despite low sequence similarity, hMed25-ACID also interacts with the plant-specific Dreb2a transcriptional activator protein. We have used GST pull-down-, surface plasmon resonance-, isothermal titration calorimetry and NMR chemical shift experiments to characterize interactions between Dreb2a and VP16, with the hMed25 and aMed25-ACIDs. We found that VP16 interacts with aMed25-ACID with similar affinity as with hMed25-ACID and that the binding surface on aMed25-ACID overlaps with the binding site for Dreb2a. We also show that the Dreb2a interaction region in hMed25-ACID overlaps with the earlier reported VP16 binding site. In addition, we show that hMed25-ACID/Dreb2a and aMed25-ACID/Dreb2a display similar binding affinities but different binding energetics. Our results therefore indicate that interaction between transcriptional regulators and their target proteins in Mediator are less dependent on the primary sequences in the interaction domains but that these domains fold into similar structures upon interaction.

## Introduction

Mediator is a multi-protein transcriptional co-regulator complex which is conserved in eukaryotes [1]–[3]. It acts as a bridge by transmitting signals from promoter-bound transcription regulators (activators and repressors) to the general RNA polymerase II (Pol II) machinery to regulate transcription of protein encoding genes [4], [5], but its function at the molecular level is still elusive. Mediator is structurally dynamic and has a high conformational flexibility which depends on intrinsically disordered regions within some of its protein subunits [6], [7]. These regions are prone to fold upon interaction with transcriptional regulators which induces structural changes in Mediator required for propagation of regulatory signals to the Pol II transcription machinery [8]–[10]. Mediator is evolutionary conserved from yeast to humans but individual Mediator subunits have diverged and some of them share only modest sequence homologies between different organisms [1], [2], [11]. In addition, the number of Mediator subunits differs between organisms, from 25 in yeast to 29 and 34 in humans and plants, respectively. The higher number of subunits in higher eukaryotes is likely related to the increased complexity of transcription regulation in multicellular organisms [2], [12]–[15].

Med25 is one of the few Mediator subunits that are specific to higher eukaryotes and human Med25 (hMed25) has been shown to interact with several transcription factors involved in different cellular processes, including retinoid signaling by RARα, chondrogenesis by SOX9, insulin secretion in pancreatic cells by HNF4α, cellular growth and differentiation by PEA3 subfamily members and endoplasmic reticulum stress response by ATF6α [16]–[19]. A specific ACtivator Interaction Domain (ACID) in hMed25 has been shown to interact with the Herpes simplex virus type 1 VP16 protein and the Varicella-zoster virus protein IE62, which activate transcription of viral genes [20]–[23]. The structure of the hMed25-ACID (residues 394–543) has been solved by NMR and it comprises seven β -strands forming a β-barrel flanked by three helices [24], [25]. The interaction between hMed25-ACID and VP16 has been studied in detailed and the VP16 binding site in the ACID has been defined [20], [22]. In addition to binding to the hMed25-ACID, the VP16 transcription activation domain (TAD) has been shown to interact with several general transcription factors, including TFIIA, TFIIB, TFIIF, TFIIH, TBP, hTAF_II_31/hTAF_II_32 (Taf9), Med15 and Med17 to activate immediate early genes during lytic infection [20], [26]–[34]. The VP16-TAD is composed of two subdomains, one N-terminal including residues 412–452 and one C-terminal which includes residues 452–490, which function independently and complementary [22], [31], [35], [36]. Similar to several other TADs (*e.g*. p53), the VP16-TAD is unstructured in its free, unbound state, but adopts an α-helical conformation upon binding to its target proteins [30], [40]. Within the two subdomains, the formation of α-helical segments has been located to residues 429–450 and 465–488 [37]. In the N-terminal TAD, a nine-amino acid sequence (DFDLDMLGD) has been identified as playing a key role for VP16 transcription activity [35], [41]. In addition, this nine-amino acid motif has also been identified in several other transcription factors and it is proposed to bind to a common co-factor. This is the case for VP16, p53, HSF1, NF-kB and NFAT1, which all contain this motif and have been shown to interact with the TAF_II_31 (TAF9) protein [42].

The *A.thaliana* Med25 (aMed25) was originally identified as *PHYTOCHROME AND FLOWERING TIME 1* (*PTF1*), which function in a phyB pathway to regulate the expression of the *FLOWERING LOCUS T* (*FT*) in response to suboptimal light conditions [43]–[45]. More recently, *PFT1* was identified as Med25 and has been shown to be involved in regulation of jasmonate (JA) signaling and different stress response pathways [12], [46], [47]. Med25 interaction with specific transcription factors results in both positive and negative regulatory effects. MYC2 interaction with Med25 is involved in activation of JA-responsive gene expression; while Med25 interaction with ABI5 had a negative regulatory effect on regulation of Abscisic acid mediated gene expression, [48]. We have previously shown that the aMed25-ACID interacts with three transcription factors; the Dehydration responsive element binding protein 2A (Dreb2a), the Myb-like transcription factor and the zinc finger homeodomain 1 protein [46]. Dreb2a interacts with cis-acting dehydration-responsive promoter elements and activates genes involved in drought- and salt stress responses [49], [50]. The transcription activation domain of Dreb2a has been localized to residues 254–335 [51] but the minimal domain required for interaction with aMed25-ACID in yeast 2-hybrid assays was localized to residues 168–254 [46] and is thus distinct from the Dreb2a TAD. The target for the Dreb2a activation domain is therefore likely to be another, yet not identified Mediator subunit. In a previous study we found that a fragment of Dreb2a (residues 168–335; Dreb2a_168–335_), which includes both the TAD and the Med25 interaction domain is unstructured in the free state and was required to bind aMed25-ACID with a high affinity *in vitro* [52]. In the same study we also showed that hMed25-ACID was able to interact with Dreb2a, which is remarkable in two aspects. First, the ACIDs from *A.thaliana* and human share low sequence similarity (16%) and secondly, Dreb2a is a plant-specific transcription factor belonging to the large AP2/ERBP (ethylene responsive element binding protein) transcription factor family [53]–[55].

In the present study we use GST pull downs- and surface plasmon resonance (SPR) experiments to show that the VP16-TAD interacts with the aMed25-ACID. Furthermore, using NMR chemical shift perturbation (CSP) and isothermal titration calorimetry (ITC) combined with available information about the well-studied interaction between hMed25-ACID and VP16-TAD, we show that the two unrelated transcription factors Dreb2a and VP16 interact with overlapping regions in the ACIDs of both human and plant Med25. Our results suggest that the Med25-ACIDs from human and *A.thaliana* retain a conserved structure and function regarding activator binding, despite a low level of homology in primary sequence. This highlights the importance of studying cross-species interactions between transcriptional regulators and their target proteins.

## Results

### aMed25-ACID interacts with the Herpes Simplex virus VP16 transcription factor

**Figure 1:**
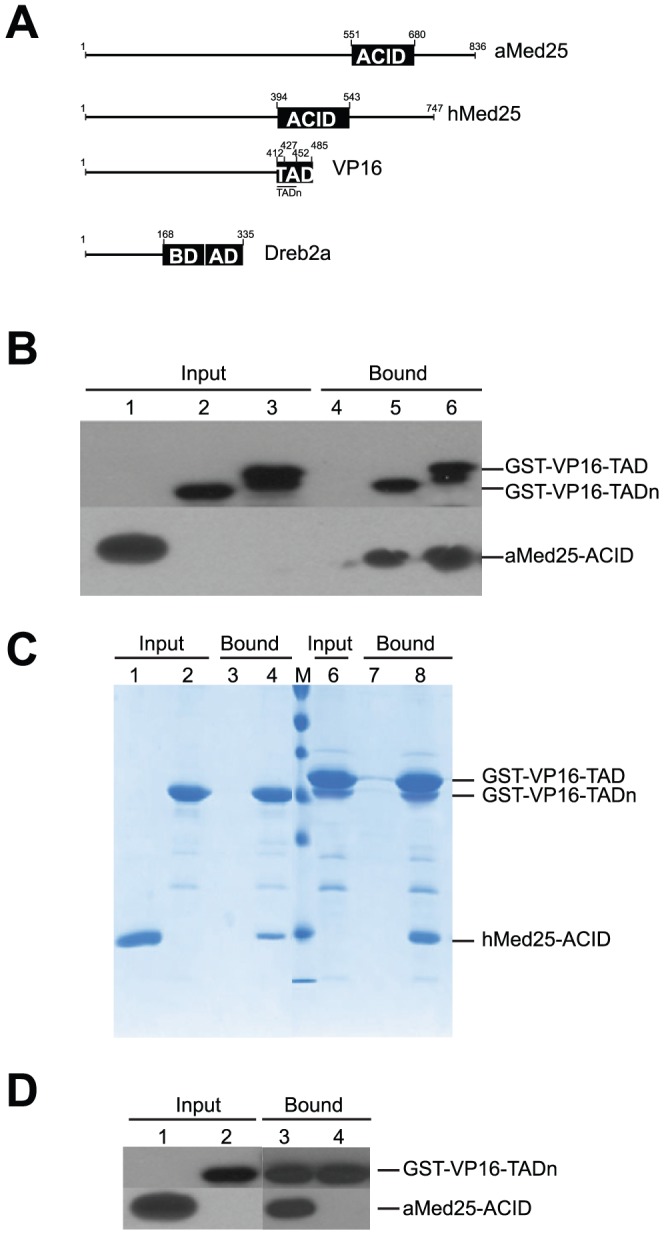
*A.thaliana* and human Med25-ACIDs interacts with the Herpes Simplex virus VP16 transcription factor. **A**) Illustration of the protein domains used in this study. ACID, activator interaction domain. TAD and AD, transcription activation domains. BD, *A.thaliana* Med25-ACID-binding domain. **B**) GST pull-down assay using purified recombinant proteins. aMed25-ACID was incubated with glutathione beads pre-bound to GST-VP16-TADs and the proteins were visualized by immunoblotting with anti-GST and anti-Med25 antibodies. Lane 1, aMed25-ACID (input); Lane 2, GST-VP16-TADn (input); Lane 3, GST-VP16-TAD (input). Lane: 4, aMed25-ACID (bound); Lane 5, aMed25-ACID + GST-VP16-TADn; Lane 6, aMed25-ACID + GST-VP16-TAD (bound). The input lanes represent 25% of the load used for each pull-down experiment. **C**) GST pull-down assay using recombinant proteins. GST-VP16-TADs were pre-bound to glutathione beads and incubated with hMed25-ACID (18 kDa). Proteins were separated on a 15% SDS-PAGE and stained with Coomassie blue. Lane 1, hMed25-ACID (input); Lane 2, GST-VP16-TADn (input); Lane 3, hMed25-ACID (bound); Lane 4, hMed25-ACID + GST-VP16-TADn (bound); Lane 6, GST-VP16-TAD (input); Lane 7, hMed25-ACID(bound); Lane 8, hMed25-ACID + GST-VP16-TAD (bound). **D**) GST pull-down assay to study competition between binding of Dreb2a_168-335_ and VP16-TAD to aMed25-ACID. Dreb2a_168-335_ and aMed25 were pre-incubated and then added to VP16-TADn bound to GST-beads. Lane 1, aMed25-ACID (input); Lane 2, GST-VP16-TADn (input); Lane 3, aMed25-ACID + GST-VP16-TADn (bound), Lane 4, aMed25-ACID pre-incubated with Dreb2a_168-335_ + GST-VP16-TADn (bound). The input lanes represent 25% of the load used for each pull-down experiment.

Previous studies have shown that hMed25-ACID interacts with the VP16-TAD [24], [25], [36], [56] and we have previously reported that hMed25-ACID unexpectedly interacts with the plant-specific Dreb2a transcription factor, even though hMed25-ACID and aMED25 show very low sequence homology. In order to study if also aMED25-ACID and VP16 can interact, we carried out binding experiments between the aMed25-ACID and the VP16-TAD using pull-downs and SPR. In our experiments we used two constructs of the GST-VP16-TAD protein to test the binding properties of the different VP16 subdomains. One construct contained the N-terminal functional subdomain (TADn) comprising residues 412–452. The second construct (VP16-TAD) contained both subdomains (residues 427–485; **Figure 1A**). GST pull-down experiments were carried out with the aMed25-ACID, but also with the hMed25-ACID as a positive control (**Figures 1B and 1C**). Western blotting using antibodies against GST and aMed25-ACID revealed binding of aMed25-ACID with both VP16-TAD-constructs (**Figure 1B**). Next, we tested if the interaction between aMed25-ACID and the VP16 TADs could be inhibited by the *A.thaliana* transcription factor Dreb2a, which we have previously shown to interact with the aMed25-ACID [46], [52]. For these pull-down experiments we used a 6 x his-tagged, C-terminally extended domain of Dreb2a, which comprised both the aMed25-binding domain (BD; 168–253) and the activation domain (AD; 254–335) (**Figure 1A**). We carried out the binding reactions as described above, but this time aMed25-ACID and Dreb2a_168-335_ were pre-incubated together before they were added to GST-VP16-TADs pre-bound to glutathione beads. Proteins bound to the beads were identified by Western blotting using anti-GST and anti-Med25 antibodies. As shown in **Figure 1D**, aMed25-ACID did not bind to the VP16-TADn when it had been pre-incubated with Dreb2a_168-335_. Similar results were obtained when using VP16-TAD (data not shown). The finding that Dreb2a_168-335_ interferes with the binding of aMed25-ACID to the VP16-TAD indicates that both Dreb2a and VP16 interact with overlapping regions on the aMed25-ACID.

**Figure 2:**
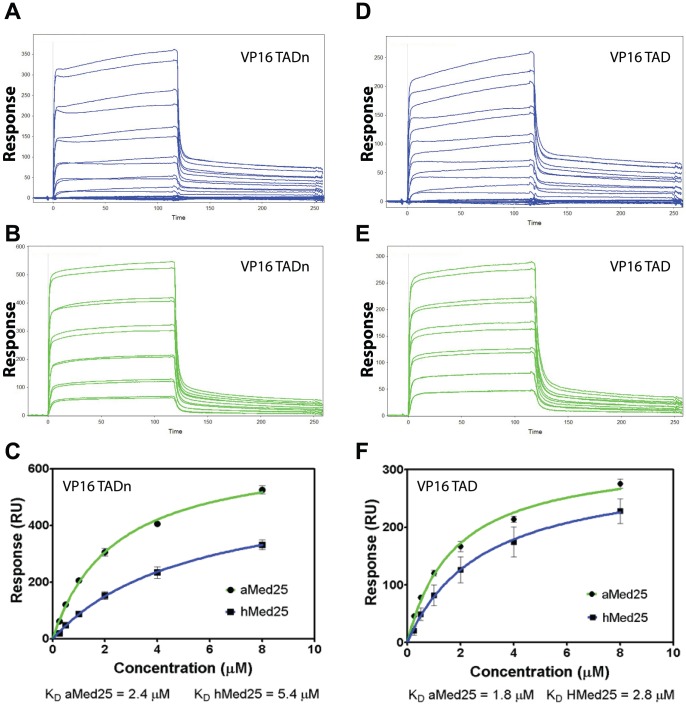
Binding kinetics for VP16-TADn and VP16-TAD to *A.thaliana* and human Med25-ACIDs. **A**) SPR sensogram for association and dissociation of hMed25 to GST-VP16-TADn. **B**) SPR sensogram for association and dissociation of aMed25 to GST-VP16-TADn. **C**) Binding curves plotted from the sensograms in A and B. The dissociation constants (K_D_) are indicated. **D**) SPR sensograms for association and dissociation of hMed25 to GST-VP16-TAD. **E**) SPR sensograms for association and dissociation of aMed25 to GST-VP16-TAD. **F**) Binding curves plotted from the sensograms in D and E. The dissociation constants (K_D_) are indicated. Green curves represent aMed25 and blue curves represent hMed25.

The interactions between different Med25-ACID and VP16-TAD proteins were further analyzed using SPR. The GST-VP16-TAD and GST-VP16-TADn subdomains were immobilized on a CM5 sensor chip using amine coupling and binding was assessed for both the hMed25-ACID (as positive control) and the aMed25-ACID. The sensogram profiles were similar for all four interactions, displaying both rapid association and dissociation kinetics. The binding kinetics was obtained by monitoring sensograms with increasing concentrations of Med25-ACID proteins injected to the immobilized VP16-TAD subdomains (**Figure 2A**–**F**). The dissociation constants (K_D_) were calculated to 2.4 µM and 1.8 µM for the aMed25-ACID/VP16-TADn and the aMed25-ACID/VP16-TAD interactions, respectively. The K_D_ values obtained for the hMed25-ACID/VP16-TADn (5.4 µM) and the hMed25-ACID/VP16-TAD (2.8 µM) interactions were in the similar range. Furthermore, the K_D_ value for interaction between the hMed25-ACID and the VP16-TADn that we obtained in our experiments is comparable to those previously reported (1.6 µM) by Wagner et al., using ITC [56].

### NMR-studies of interactions between Dreb2a and hMed25-ACID or aMed25-ACID

**Figure 3:**
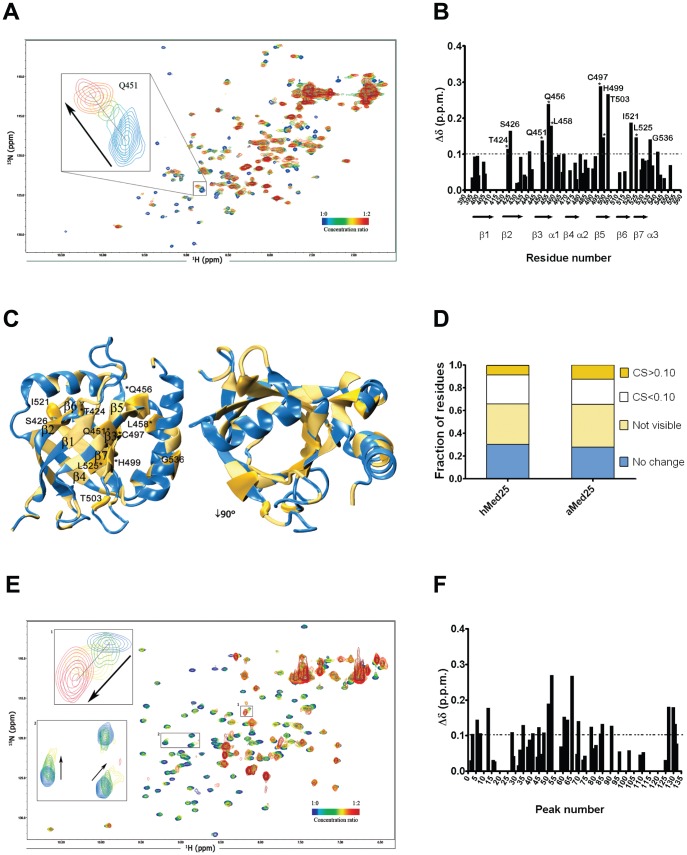
NMR studies of interactions between Dreb2a and hMed25-ACID or aMed25-ACID. **A**) ^1^H,-^15^N HSQC spectra of hMed25-ACID in the absence (blue) and in presence of 0.2 (cyan), 0.4 (green), 0.6 (yellow), 1 (orange) or 2 equivalents (red) of Dreb2a_168-335_. Inset shows an intermediate exchange shift of a resonance corresponding to residue Q451 with a curved-like shift. **B**) Chemical shift changes (Δδ) obtained from NMR experiments shown in (A) plotted against the residue number of hMed25-ACID. The position of the seven β-strands and three α-helices is indicated under the residue numbers. Residues undergoing major chemical shift changes (Δδ>0.10 ppm) are labeled with the respective residue number and asterisks indicate residues which have been previously reported to be part of the interaction surface with VP16-TAD [36], [56]. Dotted line corresponds to the threshold value of 0.10 (two chemical shift standard deviations). **C**) NMR structure of hMed25-ACID (PDB ID 2XNF) [36]. Residues undergoing significant chemical shifts upon addition of Dreb2a_168-335_ from figure (B) are enlarged and highlighted in gold and labeled with residue name and number. Resonances which are broadened beyond detection by interaction with Dreb2a_168–335_ are colored in light yellow. The seven β-strands are indicated in the left view. **D**) Comparison of the fraction of peaks which are affected to differing degrees by interaction of Dreb2a_168–335_ with Med25-ACID proteins, based on spectra presented in figure 3A and 3E. Color coding indicates peaks which are unaffected (blue), broadened beyond detection (light yellow), Δδ >0.1 (gold), Δδ <0.1 (white). **E**) ^1^H,-^15^N HSQC spectra of aMed25-ACID in the absence (blue) and in presence of 0.2 (green), 0.5 (yellow), 1 (orange) or 2 equivalents (red) of Dreb2a_168–335_. Inset 1 shows a cross-peak illustrating fast exchange with a curved-like shift. Inset 2 shows fast exchange shifts of two resonances (indicated by arrows). **F**) Similar plot as (B) but using data obtained from NMR experiments showed in (E). Δδ was plotted against the number of peaks corresponding to the number of residues of aMed25-ACID. No peak assignment is available for aMed25-ACID, therefore an identification of residues undergoing significant chemical shift changes is not possible.

We have previously shown that both hMed25-ACID and aMed25-ACID interact with the *A.thaliana* Dreb2a transcription factor [52]. Since the hMed25 ACID structure is known and its binding site for VP16-TAD is defined [24], [36], [56], we performed NMR CSP experiments in order to map the Dreb2a binding site(s) in hMed25 and to explore if the hMed25-ACID binding interface(s) for VP16-TAD and Dreb2a overlaps. NMR titration experiments were performed using isotope-labelled ^15^N hMed25-ACID and unlabeled Dreb2a_168–335_. A series of two-dimensional (2D) ^1^H,-^15^N heteronuclear single quantum-coherence (HSQC) spectra was recorded using ^15^N-labeled hMed25-ACID in free form and in the presence of different concentrations of Dreb2a_168–335_ (molar ratios of hMed25-ACID:Dreb2a_168–335_: 1∶0.2; 1∶0.4; 1∶0.6; 1∶1; 1∶2). Chemical shift assignments of hMed25-ACID were obtained from the Biological Magnetic Resonance Data Bank (BMRB, ID 17139). The chemical shift changes were analyzed at the 1∶1 ratio because the affected peaks were either undetectable or showed curved chemical shift changes in the ^1^H, ^15^N plane at higher concentrations of Dreb2a_168–335_ (**Figure 3A**, inset). The changes in the chemical shifts of the amide resonances of hMed25 upon binding to Dreb2a_168–335_ (1∶1 ratio) were plotted against the residue number in **Figure 3B** where the positions of the seven β-strands and three α-helices that comprise hMed25-ACID are indicated. An adjusted chemical shift value (Δδ) was calculated according to Δδ  =  [(ΔδH)^2^ + (0.2ΔδN)^2^]^1/2^ in order to weight contributions of ^1^H and ^15^N [57]. A threshold value was set based on two times the standard deviation σ (2σ = 0.10) [58]. Residues that displayed major chemical shifts (Δδ >0.10 ppm) in the ^1^H,-^15^N HSQC spectra were T424, S426, Q451, Q456, L458, C497, H499, T503, I521, L525 and G536. The largest effects observed were located to strands β3 and β5, which along with the other five β-strands form a β-barrel which contains a hydrophobic pocket (**Figure 3C**).

During the CSP experiments we observed several interesting features. First, we found that the hMed25-ACID residues that underwent chemical shift changes upon addition of Dreb2a were in the fast- and intermediate exchange range on the NMR time scale. In the fast exchange range, peaks did not change significantly in intensity and the shifts occurred continuously during the titration. In the intermediate exchange range (the majority of the chemical shifts above 0.10 belong to this category) the intensity of the peaks decreased and showed line-broadening while the chemical shifts changed from an unbound to a bound state (**Figure 3A**, inset). Second, ∼35% of the peaks disappeared already at the first titration ratio (1∶0.2) (**Figure 3A, D**), indicating that they are directly or indirectly involved in the binding event. However, the disappearance of these NH at this low molar ratio of Dreb2a_168–335_ indicates that they are in an intermediate exchange regime. This prevented us from obtaining information about these residues, although they might be important for complex formation, because we were unable to add Dreb2a_168–335_ at high enough concentrations due to problems with its solubility and stability. Third, we noticed that some cross-peaks had a tendency to shift in a curved-like manner (**Figure 3A** inset) which might result from secondary binding effects. Such secondary binding effects might include weak or non-specific binding of Dreb2a_168–335_ on additional sites on hMed25-ACID, or formation of higher-order complexes. It is likely that these effects are caused by the activation domain of Dreb2a, which is unstructured and potentially forms transient structural segments upon interaction with hMed25-ACID, a feature that has previously been reported for transcriptional activators upon interaction with its targets [39], [59]–[61]. For example the VP16-TAD forms helical segments upon interaction with Tfb1, PC4 and hTAF_II_31 [30], [37], [38], [60]. Such potential helical segments can also be predicted to form in Dreb2a (**Figure S1**). Finally, we mapped the significant chemical shifts and the residues that were undetectable already at the first titration point onto a ribbon diagram of the hMed25 NMR structure (PDB ID 2XNF) [36] (**Figure 3C**). From this diagram we can observe that the β-barrel that forms the hydrophobic pocket in hMED25-ACID is the most affected during ligand titration. This hydrophobic pocket has previously been described to play an important role in the hMed25-ACID/VP16-TAD interaction [56]. Interestingly, some residues that display significant chemical perturbations in our titration experiments (T424, Q451, Q456, L458, C497, H499, L525) have been described to constitute part of the interaction surface with VP16-TAD [24], [36], [56]. Q451 has been reported to have a critical role for the interaction with VP16-TADn since a mutation of Q451 to a glutamic acid prevents binding [56]. Moreover, as in the case for the hMed25-ACID/VP16-TAD interaction described by other studies [36], [56], we also observed that a large interaction surface centered on the inside or core of the β-barrel in the hMed25-ACID is affected upon binding to Dreb2a (**Figure 3C**). In summary, our results suggest that the ligand binding interface(s) in hMed25-ACID that participate in interactions with VP16-TAD and Dreb2a_168–335_ are similar or overlapping, and that the formation of hMed25-ACID/VP16-TAD and hMed25-ACID/Dreb2a_168–335_ complexes give rise to similar conformational rearrangements.

Similar NMR CSP experiments were performed using ^15^N-labeled aMed25-ACID together with unlabeled Dreb2a_168–335_ or full-length Dreb2a (Dreb2a_f.l._). 2D ^1^H,-^15^N HSQC spectra of aMed25-ACID were recorded in free form and in the presence of different molar ratios of aMed25-ACID and Dreb2a_f.l._ (aMed25-ACID: Dreb2a_f.l._; 1∶0.2; 1∶0.5; 1∶1; 1∶2). In this titration, a majority of the NMR signals disappeared with increasing concentrations of Dreb2a (**Figure S2**). Due to this fast transition, the rate at which the components of the complex exchanged between the free and bound state was difficult to analyze. Some signals shifted and decreased in intensity (**Figure S2**, inset 1), indicating fast exchange. Other signals decreased in intensity without shifting, most likely due to strong line broadening in the intermediate exchange regime (**Figure S2**, inset 2). In theory, broadened signals should reappear if the equilibrium can be shifted fully towards the complex. However, we were unable to observe such new signals because the fraction of complex remained small due to the problems with solubility and stability, which made it impossible to use Dreb2a_f.l._ at higher concentrations.

In the NMR titration using Dreb2a_168–335_ (19 kDa) we also observed that several resonances were strongly affected (loss of intensity) by the addition of Dreb2a_168–335_ at the 1∶1 ratio **(** **Figure 3E** **)**. However, the number of detectable NMR signals of aMed25-ACID was higher compared to the titration using the Dreb2a_f.l._ protein. During the titration experiment, we observed shifts of some aMed25-ACID resonances upon addition of increasing concentrations of Dreb2a_168–335_. These chemical shifts correspond to an intermediate and fast exchange (**Figure 3E** **,** inset 1,2) and some of them showed similar curved-like types of shifts (**Figure 3E** **,** inset 1) reminiscent of to those observed in the hMed25-ACID/Dreb2a_168–335_ titration experiment (**Figure 3A**). The structure of aMed25-ACID has not been solved, which makes it difficult to map the interaction site(s) precisely. However, by plotting the chemical shifts difference (Δδ) for all aMed25-ACID amide groups (**Figure 3F**), we found that the number of significant shifts was comparable to those obtained in the hMed25-ACID/Dreb2a_168–335_ experiment (compare **Figures 3B and 3F**). In addition, by comparing the amount of unaffected and undetectable residues upon titration with increasing amount of Dreb2a with the results from the hMed25-ACID experiment, we observed a similar behavior for both hMed25-ACID and aMed25-ACID (**Figure 3D**). This comparison suggests that hMed25-ACID and aMed25-ACID undergo similar conformational changes upon interaction with Dreb2a_168–335_. Based on the secondary structure prediction for aMed25-ACID (**Figure S3**), which indicates a similar content of secondary elements as the hMed25-ACID, and our results showing that hMed25-ACID and aMed25-ACID behave in a comparable manner upon interaction with Dreb2a, we speculate that the ACID domains from human and *A.thaliana* Med25 display a similar interaction surface which can adopt a similar structure upon interaction with Dreb2a.

### Complex formation of Med25-ACID proteins and Dreb2a studied by ITC

**Figure 4:**
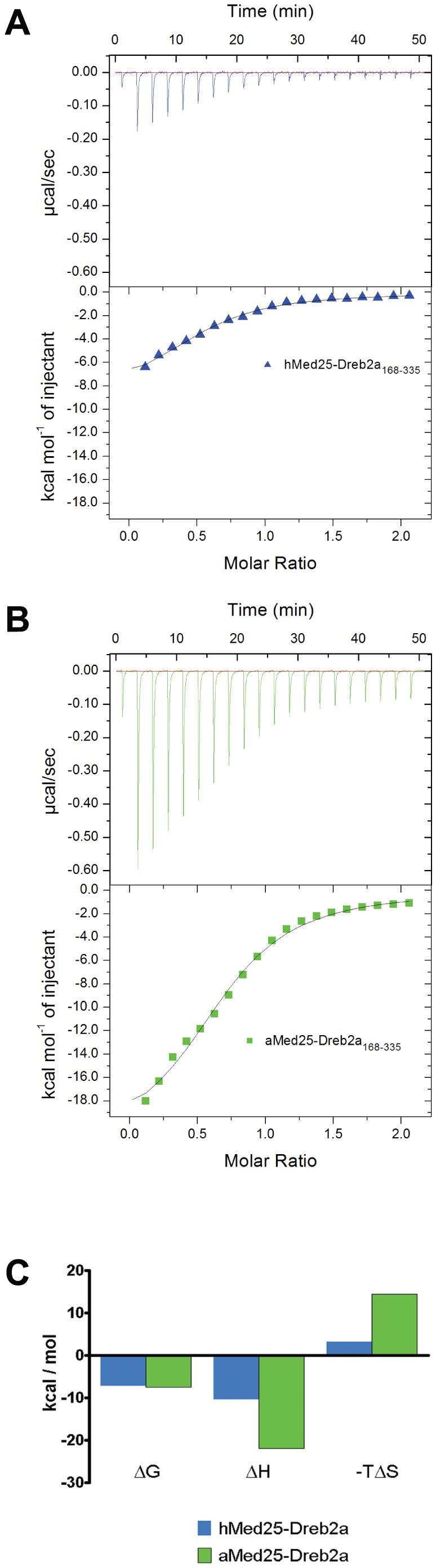
Complex formation of Med25 and Dreb2a studied by ITC. **A**) ITC profile corresponding to the binding of hMed25-ACID/Dreb2a_168–335_ (blue, triangles). **B**) ITC profile corresponding to the binding of aMed25-ACID/Dreb2a_168–335_ (green, squares). 200 µM Dreb2a_168–335_ (in the syringe) was titrated into 20 µM hMed25-ACID (A) or 20 µM aMed25-ACID (B) in the reaction chamber at 25°C. The plots in the lower panels in A and B are integrated heats from raw data (upper panels) as a function of the molar ratio of Dreb2a/Med25-ACID. The binding curves were fitted using a single site binding model (Origin, MicroCal). The thermodynamic data obtained from the fitting are presented in figure (C) and Table 1. **C**) Histograms of the binding energetics from the two binding events in (A and B) showing the differences in enthalpic and entropic contributions from each complex formation. hMed25-ACID/Dreb2a_168–335_ (blue) and aMed25-ACID/Dreb2a_168–335_ (green). Change in free binding energy (ΔG), change in binding enthalpy (ΔH) and change in entropy factor (ΔTS).

To obtain further insights into the binding mechanisms between hMed25-ACID and aMed25-ACID with Dreb2a, we preformed ITC experiments. 20 µM Med25-ACID and 200 µM Dreb2a_168–335_ were used for these titrations. The calorimetry data was evaluated using the standard model of bimolecular complex formation for single-site binding. We obtained thermodynamic data from the binding curve-fittings as shown in Table 1. Based on the obtained affinity constants (K_A_), the K_D_ values were calculated to 5.4 µM and 3.0 µM for the hMed25-ACID/Dreb2a_168–335_ and the aMed25-ACID/Dreb2a_168–335_ interactions, respectively. The binding free energy values obtained for both the hMed25-ACID/Dreb2a_168–335_ and aMed25-ACID/Dreb2a_168–335_ interactions, were small (−7 kcal/mol), which is characteristic for weak and transient protein-protein interactions, such as those typically observed between transcriptional regulators and their target proteins [62]. Even though the binding affinities and free energies that we observed for complex formation were similar to each other (**Figure 4A, B**), the binding mechanisms differed based on the large difference between the enthalpic and entropic contributions from the formation of each of the complexes (**Figure 4C**, **Table 1**). The comparison between the binding energetics from the two different interaction events showed large differences; the aMed25-ACID/Dreb2a_168–335_ interaction was more enthalpy-driven compared to the hMed25-ACID/Dreb2a_168–335_ interaction (**Figure 4C**). The negative enthalpy change, which originates from favorable weak interactions at the protein-protein surface (i.e. hydrogen- and van der Waals bonds) was almost 2-fold larger for the aMed25-ACID/Dreb2a_168–335_ interaction compared to the hMed25-ACID/Dreb2a_168–335_ interaction. In addition, the unfavorable change in entropy, which may be caused by folding of Dreb2a_168–335_ into a more well-defined structure upon complex formation, was around 4-fold larger for aMed25-ACID/Dreb2a_168–335_ compared to the corresponding values for the hMed25-ACID/Dreb2a_168–335_ interaction (**Figure 4C**). These results suggest that the complex formation between Dreb2a_168–335_ and aMed25-ACID involves larger conformational rearrangements compared to those that occur upon binding between hMed25-ACID and Dreb2a_168–335_.

**Table 1:** Thermodynamic data extracted from ITC measurements.

	ΔG(kcal/mol)	ΔH(kcal/mol)	TΔS(kcal/mol)	KA(M^−1^)	K_D_( µM)
hMed25-Dreb2a	−7.18	−10.43	−3.24	1.84E5	5.4
aMed25-Dreb2a	−7.50	−21.94	−14.43	3.28E5	3.0

Binding parameters were determined by using a single site binding model (Origin, MicroCal). Total free binding energy change ΔG, enthalpy change ΔH, entropy factor change ΔTS, affinity constant KA and dissociation constant KD.

From our NMR results on these interactions, we deduced that binding of Dreb2a_168–335_ affects large regions in both of the Med25-ACID proteins. However, we could not directly describe the different binding mechanisms that were deduced from our ITC experiments. Similar unfavorable entropy changes were obtained in a study between the transcriptional coactivator β-Catenin and the unstructured transcription factor Lef-1 [63]. The process of binding between two or more proteins -which confers functionality-, is the result of several complementary attributes at the protein's interfaces, ranging from the amino acid composition, hydrophobicity, electrostatic interactions and hydrogen bonding. All these attributes determine the energetics of the protein-interfaces and the free energy contributions have to be kept in balance for stable complex formation. This process involves a balance between entropy and enthalpy changes where the unfavorable changes might be compensated in the regions that are not part of the binding site [64]–[67], which might explain the differences in binding energetics between hMed25-ACID/Dreb2a and aMed25-ACID/Dreb2a. Therefore, even though the plant-specific transcription factor Dreb2a is able to bind to the hMed25-ACID, it might still give rise to a different and specific functional fold upon complex-formation with the aMed25-ACID which might be required for proper functionality during activation of transcription in *A.thaliana*. Similar ITC experiments using VP16 were not carried out since GST cleavage of the fusion protein resulted in very low yields wherefore the protein concentrations required for performing ITC experiments could not be obtained.

## Discussion

We have used a series of protein-protein interaction methods to show and characterize the binding between Med25-ACID from human and *A.thaliana*, with the Herpes simplex virus VP16- and plant specific Dreb2a transcription factors. Several studies have already described the interaction between hMed25-ACID and VP16-TAD, and the binding site has been mapped [24], [36], [56]. To our knowledge, this is the first time that human herpes simplex virus VP16-TAD has been reported to interact with *A.thaliana* Med25-ACID. Our earlier findings showing that hMed25-ACID interacts with the *A.thaliana* transcription factor Dreb2a [52], were here studied in more detail using NMR and ITC. Our NMR experiments showed that the hMed25-ACID interaction surface for Dreb2a_168–335_ overlaps partially with the previously reported VP16-TAD binding site [24], [36], [56]. VP16 and Dreb2a are unrelated transcription factors that display an amino acid sequence identity of only 11% (**Figure S1**). Notably, we found that six amino acids in the Dreb2a sequence are identical and aligns to a region in VP16-TAD containing nine amino-acids (DFDLDMLGD), which has previously been reported as important for interaction with several regulatory proteins [41], [68]. This nine amino-acid sequence has also been identified in a range of transcription factors and might represent a novel motif which can be recognized by a common co-factor. Such is the case for VP16, p53, HSF1, NF-kB and NFAT1 which both contain the nine amino-acid sequence and interact with the general co-activator TAF9 (TAFII31) [30], [42], [69]. However, this motif is absent in the two other transcription factors, Myb-like and ZFHD1, which we identified as interacting with Med25-ACID in the same two-hybrid screen were we found Dreb2a [46]. This is not surprising, since we found that ZFHD1 and Myb-like are involved in stress-response pathways, while Dreb2a in addition is involved in regulation of light-quality pathways downstream of the PhyB receptor.

On the other hand, the human and *A.thaliana* Med25-ACID homologs share only 16% identity in amino acid sequence but their secondary structure contents appear to be highly similar (**Figure S3**). Despite the low homology, our study shows that the ACIDs from each of the human and *A.thaliana* Med25 are able to interact with both of the unrelated transcription regulators VP16 and Dreb2a *in vitro.* In addition, both transcription regulators seem to share interaction surfaces on the Med25-ACIDs. It is likely that both the transcriptional regulators and the Med25-ACIDs contribute to the recognition specificity for these particular protein-protein interactions. At one side, the VP16 and Dreb2a TADs both belong to the acidic activator family and share the nine amino-acid common motif found in several other TADs. On the other side, the ACIDs from human and *A.thaliana* show structural similarities which might provide the interaction surfaces required for binding of the transcriptional regulators. Moreover, our study provides insight into the mechanism for interaction between Dreb2a and the Med25-ACID proteins. Our NMR experiments show that a comparable fraction of both arabidopsis and human Med25-ACID residues are affected upon binding of Dreb2a. A detailed analysis of the interaction surfaces is hampered by strong line broadening probably due to secondary binding effects. These effects influence our NMR results, but are not detected in our ITC or SPR experiments, because of the higher protein concentrations used in the NMR experiments. Our ITC data indicate that the proteins have similar affinities but different binding energetics, which might result in distinct conformational rearrangements upon complex formation. The conformational changes appear to be larger for the interaction between the Med25-ACID and Dreb2a_168–335_ from *A.thaliana* relative to the human Med25-ACID and Dreb2a_168–335_. This might be relevant for proper biological function in the normal context, where the Mediator complex in *A.thaliana* needs to be triggered by interaction with Dreb2a to induce the structural rearrangements that are required to perform its function. As mentioned in the introduction, Mediator has been shown to be structurally dynamic and flexible and the binding to activators induces global structural shifts [9].Our ITC experiments showed differences in energetics when comparing Dreb2a binding to human or *A.thaliana* Med25-ACIDs. We therefore speculate that the plant transcription factor Dreb2a might not induce such structural shifts upon interaction with human Med25-ACID since these proteins originate from different species. It would be interesting to compare these results with the conformational changes that VP16-TAD would induce in *A.thaliana* Med25-ACID. However, as already mentioned such experiments could not be performed due to difficulties to achieve sufficiently high protein concentrations. Altogether, our findings that the ACID from human and plant Med25 can interact with the unrelated VP16 and Dreb2a transcription factors, suggest that even though the Mediator complex sequences have diverged rapidly during evolution, the structure and interaction properties of its subunits remain conserved.

## Materials and Methods

### Protein expression and purification

Full-length 6 x his-GB1 tagged Dreb2a (Dreb2a_f.l._), 6 x his-tagged Dreb2a_168–335_, *A.thaliana Med25_551_*_–680_ (aMed25-ACID) and 6 x his-tagged human Med25_394-543_ (hMed25-ACID) were expressed in *E. coli* BL21 pLysS cells and purified as described previously [46], [52]. Cells for expression of isotope labeled human and *A.thaliana* Med25-ACID were grown in M9 medium (22 mM Na_2_HPO_4_, 22 mM KH_2_PO_4_, 8.5 mM NaCl, 22.2 mM glucose, 1 mM MgSO_4_, 0.1 mM CaCl_2_, 29.6 µM thiamine, 40.9 µM biotin and trace elements, pH 7.4) containing 18.6 mM ^15^NH_4_Cl as the only nitrogen source. Expression was induced by addition of isopropyl β-D-1-thiogalactopyranoside (IPTG) to a final concentration of 0.5 mM at an OD_600_ of 1. The cells were then grown over night at 18°C and proteins were purified as described [52]. pETM30 vectors encoding VP16-TAD constructs (TADn: residues 412–452; VP16-TAD: residues 427-485) were kindly provided by Prof. Gerhard Wagner (Harvard Medical School, BCMP). The vectors were transfected into *E. coli* BL21 pLysS and protein expression was induced by addition of IPTG to a final concentration of 1 mM at an OD_600_ of 0.8 followed by incubation for four hours at 30°C. GST-fusion VP16-TAD proteins were purified by affinity chromatography on glutathione-sepharose 4B (GE Healthcare) following the manufacturer's instructions. All purified proteins were transferred in to buffer A (20 mM Tris/HCl pH 7.5, 150 mM Na_2_SO_4_, 0.5 mM DTT) using dialysis cassettes or spin columns (Thermo Scientific). Protein samples were concentrated using ultrafiltration spin columns (Vivaspin, Sartorius) and the protein concentrations were calculated by determining their absorption at 280 nm and by using the proteins' extinction coefficients (E_280_ M^−1^ cm^−1^: 15,470 for aMed25-ACID, 22,460 for hMed25-ACID, 66,350 for Dreb2a_f.l._, 26,930 Dreb2a_168–335_, 44,350 for GST-VP16-TAD and 42,860 for GST-VP16-TADn). The protein concentration for VP16-TADn, which lacks tyrosines and tryptophanes, was calculated based only on the extinction coefficient for GST (E_280_ = 42,860 M^−1^ cm^−1^).

### GST pull-down experiments

For each reaction, 5 µM GST-VP16-TAD were bound to 20 µl glutathione-sepharose beads which had been pre-washed in an equilibration buffer (20 mM Tris/HCl pH 7.5, 150 mM Na_2_SO_4_) for one hour at 4°C. The beads were washed with equilibration buffer and pre-bound GST-VP16-TAD proteins were incubated with 5 µM of human Med25 or *A.thaliana* Med25 at 4°C. For the competition assays, 5 µM each of the *A.thaliana* Med25-ACID and 5 µM Dreb2a_168–335_ proteins were pre-incubated for 1 hour at 4°C. Unbound proteins were collected in the flow-through and proteins bound to the beads were washed three times with 1 ml of ice-cold equilibration buffer. Bound proteins were the directly eluted by boiling in sample buffer. 2.5% of the bound proteins from each reaction were separated on 15% SDS-PAGE and proteins were identified by Western blotting using anti-GST (Sigma) and anti-aMed25-ACID (Agrisera) antibodies.

### Surface Plasmon Resonance

The SPR experiments were performed using a Biacore 3000 instrument (GE Healthcare). GST-VP16-TADs were immobilized onto a CM5 sensor chip by amine coupling according to the manufacturer's instructions (GE Healthcare). About 500 resonance units (RU) of GST-VP16-TADs were immobilized using 10 mM sodium acetate buffer pH 4.5 and HBS-EP as running buffer (10 mM HEPES pH 7.4, 150 mM NaCl, 3 mM EDTA). Kinetics experiments were carried out by injecting decreasing concentrations of hMed25-ACID and aMed25-ACID (8 µM with 2-fold dilutions down to 0.25 µM) at a flow rate of 20 µl/min (running buffer: 20 mM Tris/HCl pH 7.5, 150 mM Na_2_SO_4_). Experiments were performed in duplicates and data was analyzed using the Scrubber2 (BioLogic software) and illustrated using the Graph Pad software.

### NMR chemical shift perturbations experiments

NMR titration experiments were performed on a Bruker Avance III 850 MHz spectrometer equipped with a ^−1^H, ^13^C, ^15^N TCI cryoprobe. 2D HSQC spectra were recorded for ^15^N-labeled hMed25-ACID or ^15^N-labeled aMed25-ACID (150 µM) in the absence and in the presence of increasing concentrations of unlabeled Dreb2a_168–335_ (molar ratio hMed25-ACID:Dreb2a_168–335_: 1∶0.2; 1∶0.4; 1∶0.6; 1∶1; 1∶2) (molar ratio aMed25-ACID:Dreb2a_168–335_: 1∶0.2; 1∶0.5; 1∶1; 1∶2). For the NMR titration with Dreb2a_f.l_, 2D HSQC spectra of 25 µM ^15^N-labeled aMed25-ACID were recorded in its free state and in the presence of 12.5, 25 and 50 µM Dreb2a_f.l._. The sample for each titration point was prepared independently and used at these low concentrations since Dreb2a_f.l._ precipitates at higher concentrations. All experiments were recorded at 25°C in 20 mM Tris/HCl pH 7.5, 150 mM Na_2_SO_4_, 0.5 mM DTT, 10% (v/v) D_2_O. Data were processed using Topspin 3.0 software (Bruker) and analyzed using the NMRviewJ software. Assignments of backbone chemical shifts of human Med25-ACID were obtained from the Biological magnetic Resonance bank with the accession number 17139 (www.bmrb.wisc.edu). Chemical shift changes of ^1^H and of ^15^N were weighted using the formula Δδ  =  [(ΔδH)^2^ + (0.2ΔδN)^2^]^1/2^ [57] to take the chemical shift ranges of ^1^H and ^15^N into account.

### Isothermal titration calorimetry

Binding experiments were performed using an auto ITC_200_ (MicroCal, GE Healthcare) at 25°C. Protein concentrations in the reaction chamber were 20 µM of hMed25-ACID or 20 µM of aMed25-ACID. 200 µM Dreb2a_168–335_ was used in the syringe for the titration. For the control experiment, Dreb2a was titrated into buffer A (20 mM Tris/HCl pH 7.5, 150 mM Na_2_SO_4_, 0.5 mM DTT). For each experiment, we performed 19 automated injections of 2 µl each with 150 s intervals between each injection and with a stirring speed of 1000 rpm. The titrations were repeated twice. Calorimetric data were plotted and fitted using the standard single-site binding model (Origin, MicroCal).

## Supporting Information

Figure S1 **Sequence alignment of the TADs from Dreb2a and VP16.** Asterisks indicate identical amino acids. The regions in VP16-TAD which have propensity to form α-helices upon interaction with its target are indicated (435–450 and 465–485) [37]. The black box indicates the region of the nine-amino-acid sequence that has previously been reported as important for interaction of VP16-TAD with several co- factors [41], [66] and which also has been identified in a range of transcription factors such as VP16, p53, HSF1, NF-kB and NFAT1 [30], [42], [67]. The Dreb2a sequence has six identical amino acids that align to that region in VP16-TAD (DFDLDMLGD).
(EPS)

Figure S2 **Interaction between aMed25-ACID and Dreb2a_f.l._** 2D ^1^H,-^15^N HSQC spectra of aMed25-ACID in the absence (blue) and in the presence of 0.2 (green), 0.5 (yellow), 1 (orange) and 2 equivalents (red) of Dreb_f.l._ Fast transition between the free and bound state could not been analyzed. Inset 1, modest chemical shift perturbation of a resonance upon addition of 0.5 equivalents Dreb2a_f.l._ which was undetectable at higher ligand concentrations. Inset 2, example of a cross-peak decreasing in intensity upon addition of Dreb2a_f.l._ (bottom left) which might indicate slow exchange and an unaffected cross-peak (upper right).
(EPS)

Figure S3 **Sequence alignment of the** ***A. thaliana*** **and human Med25-ACID.** Asterisks indicate identical amino acids. Secondary structure content of human Med25-ACID extracted from PDB ID 2XNF and secondary structure prediction for *A. thaliana* Med25-ACID using Jpred 3 server. Alpha helical content is highlighted in pink (H) and β-strands are highlighted in blue (B). Human Med25-ACID contains 7-strands and 3 α-helices while *A. thaliana* Med25-ACID is predicted to contain 7-strands and 2 α-helices.
(EPS)
